# Endothelial Expression of Scavenger Receptor Class B, Type I Protects against Development of Atherosclerosis in Mice

**DOI:** 10.1155/2015/607120

**Published:** 2015-10-04

**Authors:** Boris L. Vaisman, Tatyana G. Vishnyakova, Lita A. Freeman, Marcelo J. Amar, Stephen J. Demosky, Chengyu Liu, John A. Stonik, Maureen L. Sampson, Milton Pryor, Alexander V. Bocharov, Thomas L. Eggerman, Amy P. Patterson, Alan T. Remaley

**Affiliations:** ^1^Lipoprotein Metabolism Section, Cardiovascular-Pulmonary Branch, National Heart, Lung, and Blood Institute, National Institutes of Health, Bethesda, MD 20892, USA; ^2^Division of Diabetes, Endocrinology, and Metabolic Diseases, National Institute of Diabetes and Digestive and Kidney Diseases, National Institutes of Health, Bethesda, MD 20892, USA; ^3^Transgenic Core, National Heart, Lung, and Blood Institute, National Institutes of Health, Bethesda, MD 20892, USA; ^4^Department of Laboratory Medicine, Clinical Center, National Institutes of Health, Bethesda, MD 20892, USA

## Abstract

The role of scavenger receptor class B, type I (SR-BI) in endothelial cells (EC) was examined in several novel transgenic mouse models expressing SR-BI in endothelium of mice with normal C57Bl6/N, apoE-KO, or *Scarb1*-KO backgrounds. Mice were also created expressing SR-BI exclusively in endothelium and liver. Endothelial expression of the Tie2-*Scarb1 *transgene had no significant effect on plasma lipoprotein levels in mice on a normal chow diet but on an atherogenic diet, significantly decreased plasma cholesterol levels, increased plasma HDL cholesterol (HDL-C) levels, and protected mice against atherosclerosis. In 8-month-old apoE-KO mice fed a normal chow diet, the Tie2-*Scarb1* transgene decreased aortic lesions by 24%. Mice expressing SR-BI only in EC and liver had a 1.5 ± 0.1-fold increase in plasma cholesterol compared to mice synthesizing SR-BI only in liver. This elevation was due mostly to increased HDL-C. In EC culture studies, SR-BI was found to be present in both basolateral and apical membranes but greater cellular uptake of cholesterol from HDL was found in the basolateral compartment. In summary, enhanced expression of SR-BI in EC resulted in a less atherogenic lipoprotein profile and decreased atherosclerosis, suggesting a possible role for endothelial SR-BI in the flux of cholesterol across EC.

## 1. Introduction

Removal of excess of cholesterol from macrophages in the vasculature is crucial for preventing the progression of atherosclerotic plaques [1–3]. HDL is the main lipoprotein acceptor for the removal of excess cellular cholesterol by the reverse cholesterol transport (RCT) pathway [4, 5]. Endothelial cells (EC) represent a potential barrier to HDL in reaching macrophages in the vessel wall [6, 7]. It has been shown, however, that HDL and lipid-free apoA-I are able to cross intact aortic EC monolayers from the apical to the basolateral compartment in a transcytosis process, involving ABCG1 and scavenger receptor class B, type I (SR-BI) [8, 9]. Recently, we showed that endothelial specific expression of ABCA1 in mice also enhances cholesterol efflux from EC, increases the plasma level of HDL cholesterol (HDL-C), and protects against atherosclerosis [10]. In addition to ABCA1, Panzenboeck and colleagues demonstrated that reverse cholesterol transport by HDL across the blood-brain barrier also involves SR-BI expression in brain EC [11].

SR-BI is abundantly expressed in almost all EC [8, 12] and is a key player in HDL metabolism. It is a type 1 integral plasma membrane protein and contains two transmembrane domains [13]. The extracellular loop of SR-BI can interact with a variety of lipoproteins, including HDL [13]. It participates in cholesterol efflux from cells to HDL and is also responsible for selective uptake of cholesteryl esters from HDL by hepatocytes and adrenal cells [14]. Hepatic expression of SR-BI in mice has been shown to be atheroprotective by lowering proatherogenic lipoproteins [15]. Hepatic SR-BI also lowers the plasma level of HDL-C but it does so by increasing the uptake of cholesteryl esters from HDL by the liver, thus causing an overall increase in the flux of cholesterol by the RCT pathway [16]. Extrahepatic production of SR-BI has also been shown to be atheroprotective, but it is not known which tissue or cell type accounts for this finding [17]. It has been shown that depending on the stage of atherosclerosis development, expression of SR-BI by macrophages can be either pro- or antiatherogenic [18–21]. Expression of SR-BI in endothelial cells has also been shown to be beneficial by a variety of different* in vitro* assays [22], particularly for HDL activation of endothelial NO synthase and HDL-induced angiogenesis* in vivo* [23, 24]. However, whether increased SR-BI in EC is antiatherogenic in an animal model has not been previously assessed.

To better understand the role of SR-BI in the endothelium, we created a new transgenic mouse (Tie2-*Scarb1*) expressing SR-BI in EC, using the Tie2 endothelial cell specific promoter. We also produced a new line of mice that expressed SR-BI only in EC and no other tissues or cell types, by transferring the Tie2-*Scarb1* transgene onto the* Scarb1* knockout mouse background (Tie2-*Scarb1* ×* Scarb1*-KO). In addition, mice were also created with dual expression of SR-BI exclusively in endothelium (Tie2-*Scarb1* transgene) and liver (human* SCARB1* gene under control of a liver-specific promoter) in mice with the* Scarb1* knockout background. Findings from these mice, along with* in vitro* cell culture studies, indicate that increased levels of SR-BI in EC can favorably alter the lipoprotein profile and reduce diet-induced atherosclerosis.

## 2. Materials and Methods

### 2.1. Generation of Tie2-*Scarb1* and LIV11-*SCARB1* Transgenic Mice

Full-length (1.8 kb) mouse* Scarb1* cDNA [25] (GenBank: U37799) was flanked by Not I linkers and inserted into the Not I cloning site of pSPTg.T2FpAXK, which contains 2.1 kb of the mouse EC specific mouse Tie2 promoter and a 1.6 kb Tie2 enhancer [26, 27]. Clones with correct orientation of the transgene were selected by restriction analysis with Bgl I or Nar I. After plasmid digestion with Sal I, a 5.6-kb DNA fragment containing the complete expression cassette was isolated from a 0.8% agarose gel and purified by CsCl density gradient ultracentrifugation (Beckman TL-100 table top ultracentrifuge at 95000 RPM for 24 hrs at 20°C). After dialysis against 10 mM Tris-HCl, pH 7.4, 0.1 mM EDTA, the DNA fragment was microinjected into pronuclei of fertilized eggs from C57Bl/6J females (Jackson Laboratory, ME, USA) [28].

Generation of LIV11-*SCARB1* transgenic mice was described previously [29].

Genotyping and expression analysis of the transgenic mice by real-time PCR and genotyping by traditional PCR was performed as described previously [10] and in the Supplemental Material (see Supplementary Materials available online at http://dx.doi.org/10.1155/2015/607120). The relative level of gene expression was measured by the comparative C_T_ (ΔΔC_T_) method [30], with mouse *β*-actin, 18S, or 28S rRNA genes used for normalization. The absence of contaminating DNA from transgenic mice was verified by performing no reverse transcription controls.

All breeding was done with C57Bl/6N mice (Taconic, NY, USA). The Tie2-*Scarb1* and LIV11-*SCARB1* transgenes were transferred into* Scarb1*-KO mice (Jackson Laboratory, Stock Number 3379), yielding mice that selectively synthesized SR-BI only in EC and liver, respectively. During breeding, the line of mice with the Tie2-*Scarb1* transgene on a* Scarb1*-KO background were fed a diet containing 0.5% probucol to improve their fertility [31]. Tie2-*Scarb1* was also transferred onto the apoE-KO mouse background (Jackson Laboratory, Stock Number 002052). Tie2-*Scarb1* and LIV11-*SCARB1* double transgenic mice on a* Scarb1*-KO background were generated by crossing.

Lipids were measured enzymatically, as previously described [10]. Plasma lipoproteins were fractionated by FPLC (Akta FPLC; GE Healthcare) on two Superose 6 columns in series. All animal experiments were approved by the Animal Care and Use Committee of the NHLBI (#H-0050R2). Details of animal housing, normal and high fat high cholesterol (HFHC) Paigen diet, (Harlan Teklad, TD90221) and analysis of aortic lesions were provided previously [10].

### 2.2. Western Blot Analysis

Membrane fractions were isolated from livers from 1 g of tissue per sample [32] in the presence of a protease inhibitor cocktail from Roche. Membrane pellets were resuspended in 1.0 mL Urea Lysis Buffer (8.9 M urea, 2%  *β*-mercaptoethanol (v/v), 1% NP-40 (v/v)). 20 *μ*g of liver membrane proteins was loaded onto a 4–12% Bis-Tris MOPS NOVEX gel (Life Technologies, Carlsbad, CA) for Western analysis. As a loading control, 20 *μ*g from the same aliquot was loaded side by side on the same gel for Coomassie staining (Simply Blue, Life Technologies). After electrophoresis, half the gel was used for Western blotting onto PVDF membranes (0.45 *μ*m pore size) (Immobilon-P, Millipore, Billerica, MA) and the other half was Coomassie-stained for protein. The primary antibody was a rabbit polyclonal antibody against residues 450–509 from mouse SR-BI (Novus Biologicals #NB400-101, Littleton, CO) and the secondary antibody was donkey anti-rabbit-HRP (Abcam #7083, Cambridge, MA).

### 2.3. Cell Culture Studies

Mouse peritoneal macrophages and aortic EC were isolated as described previously [10]. Immortalized mouse EC SVEC4-10 (ATCC number CRL-2181), human endothelium-derived EA.hy926 cells (ATCC number CRL-2922), and human primary aortic EC (ATCC number PCS-100-011) were grown according to supplier's recommendations. In experiments using cultured polarized EC, cells were grown on 12-mm collagen-coated Transwell inserts with 0.4 *μ*m pore PTFE membranes (Corning, Cat. no. 3493). Approximately 400,000 cells were seeded per Transwell, and cells were grown until they reached confluence, which usually took 3 days. During this period, confluence was monitored by microscopic observations and by monitoring resistance, using the EVOM resistance meter and the Endohm-12 chamber (World Precision Instruments). In all Transwell experiments, the volumes of media in the top and bottom compartments were 0.5 mL and 1.5 mL, respectively.

In experiments testing the ability of EC to acquire cholesteryl esters from HDL, the lipoproteins were labeled as described before [33] with cholesteryl oleate [1-^14^C] (American Radiolabeled Chemicals, Cat. no. 0689-50 *μ*Ci, 0.1 mCi/mL). Ultracentrifugation was performed in an Optima Max tabletop with a TL120 rotor (Beckman Coulter, Inc., USA). When EC reached confluence, the cultivation media were changed to DMEM with 0.1% fatty acid-free BSA. Radiolabeled HDL was added to the insert or bottom compartments at a final concentration of 50 *μ*g of protein per 1 mL and EC were incubated for 4 hr. Conditioned media from the apical and basolateral compartments were collected, cell monolayers were washed 2 times with Dulbecco's PBS without Ca^2+^ and Mg^2+^, and radioactive counts were then determined in media from the apical and basolateral compartments and the cell fraction. Retention of cell confluence and integrity at the end of the incubation period was monitored by measurement of the electrical resistance before and after each experiment and by microscopic observation. All analysis was done in at least two independent experiments performed in quadruplicate.

### 2.4. Immunofluorescent and Confocal Microscopy

For SR-BI visualization, we used rabbit anti-SR-BI pAB (Novus, NB400-101) diluted 1 : 100, which reacts with human and mouse SR-BI, and goat anti-rabbit Alexa568 secondary AB (Life Technologies, Rockford, IL, Cat. no. A11036). To visualize EC, rat anti-mouse CD31 antibody from BD Pharmigen, San Jose, CA, Cat. no. 550274, was used; to visualize hepatocytes, polyclonal cytokeratin 8+18 antibody was used (Fitzgerald, Acton, MA, Cat. no. 20R-CP004).

Tissue cryosections of approximately 8 *μ*m thickness were washed in cold PBS and fixed in 3.7% paraformaldehyde for 10 minutes at room temperature. Sections were then washed with PBS, permeabilized with 0.1% saponin, and blocked with 10% normal goat serum, 0.02% saponin, and 1% BSA in PBS for 1 h. After washing with PBS, sections were incubated with SR-BI primary antibody diluted 1 : 100 in PBS, 0.02% saponin, and 1% BSA overnight at 4°C. Incubation with secondary antibody was for 2 hrs with three subsequent washes in PBS for 10 minutes each. Sections then were stained with DAPI (for nuclear visualization), rinsed in PBS, mounted on glass slides with Vectashield mounting medium (Vector Laboratories, Burlingame, CA, Cat. no. H-1400), and analyzed by confocal laser-scanning immunofluorescence microscopy (Zeiss LSM 510).

For immunofluorescent analysis of SVEC4-10 cells, the cells were grown on 12-mm Transwells with 0.4 *μ*m Pore Polyester Membrane Insert (Corning, Cat. no. 3460), as described in Section 2.3, until they reached confluence, and then were washed with PBS and fixed in 3.7% paraformaldehyde for 10 minutes at room temperature. Fixed cells were washed repeatedly with PBS for 15 minutes, permeabilized with 0.1% saponin, and blocked with 10% normal goat serum, 0.02% saponin, and 1% BSA in PBS for 1 hr. After washing with PBS, cells were incubated overnight at 4°C with SR-BI primary antibody (Novus, NB400-101) diluted in PBS with 0.02% saponin and 1% BSA (final dilution 1 : 100). Incubation with secondary antibody was for 2 hrs with three subsequent washes in PBS for 10 minutes each. Cells then were stained with DAPI (for nuclear visualization) and rinsed in PBS. Membranes were cut and mounted on glass slides with Vectashield mounting medium.

Stacks of images were collected throughout the depth of cells, using a Zeiss LSM510 confocal microscope (Carl Zeiss MicroImaging, Jena, Germany) with a x63NA1.4 oil immersion objective using a 405-nm laser for DAPI and a 561-nm laser for Alexa-568. To evaluate the SR-BI localization at the apical and basolateral domains of the plasma membrane, confocal microscopy images were analyzed in 3D with Imaris software v7.7.2 (Bitplane, South Windsor, CT).

### 2.5. Statistics

Unless otherwise indicated, all data were analyzed by Student's* t*-test with GraphPad PRISM version 5.04 software. Statistically significant differences were defined as a two-tailed probability of less than 0.05. Results are presented as means ± SEM. For gene expression analysis ABI SDS 2.4 and Qiagen REST 2009 v.2.013 software were used.

## 3. Results

### 3.1. Expression Analysis of the Tie2-*Scarb1* Transgene in Comparison with Expression of Endogenous* Scarb1*

**Figure 1:**
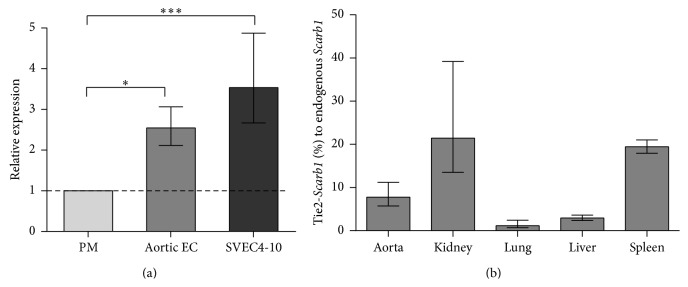
RT-PCR analysis of* Scarb1 gene* expression. (a) Expression of endogenous* Scarb1* in cultures of normal mouse aortic EC and SVEC4-10 cells relative to expression of the same gene in peritoneal macrophages. ^*∗*^ *P* < 0.04; ^*∗∗∗*^ *P* < 0.0001. (b) Expression of* Scarb1* transgene in tissues of Tie2-*Scarb1* ×* Scarb1*-KO females is shown as the percentage of the level of expression of endogenous* Scarb1* in the same tissues of normal C57Bl/6N females. *N* = 3 in each case. Results are presented as the mean ± 1 SEM.

We first evaluated the level of expression of the endogenous* Scarb1* gene in mouse EC. In primary cultures of mouse aortic endothelial cells, the* Scarb1* gene was expressed at 2.5-fold higher levels than in mouse peritoneal macrophages (*P* < 0.04, Figure 1(a)). For the mouse SVEC4-10 EC cell line, this difference was even higher, reaching 3.5-fold increase (*P* < 0.0001, Figure 1(a)). In order to demonstrate the endothelial specificity of Tie2-*Scarb1* transgene expression, we measured the level of transgenic RNA in peritoneal macrophages, isolated from Tie2-*Scarb1* ×* Scarb1*-KO mice. As previously described [10, 26, 27], the Tie2 promoter was found to be relatively cell selective for EC. Only traces of* Scarb1* mRNA were observed in the isolated macrophages; the level of* Scarb1* mRNA in normal peritoneal macrophages was more than 100-fold higher than in peritoneal macrophages from Tie2-*Scarb1* ×* Scarb1*-KO mice (*P* < 0.0002, *N* = 5, in each group).

Next, we evaluated the level of expression of the Tie2-*Scarb1* gene in different tissues of the transgenic mice. In order to eliminate interference from the endogenous* Scarb1* gene, the level of expression achieved in tissues of Tie2-*Scarb1* transgenic mice was determined after they were crossed with* Scarb1*-KO mice to produce Tie2-*Scarb1* ×* Scarb1*-KO animals. With the TaqMan assay for mouse* Scarb1*, no* Scarb1* expression was observed in* Scarb1*-KO mice (data not shown), but* Scarb1* mRNA was detected in all tissues from the Tie2-*Scarb1* ×* Scarb1*-KO mice (Figure 1(b)). When expressed as % relative to the endogenous* Scarb1* gene in normal C57Bl/6N mice the level of* Scarb1* mRNA from the transgene varied between 1 and 20%, depending on the tissue. Taking into account the EC-specificity of the expression of the transgene and the fact that EC only represent approximately 1–3% of total number of cells in most tissues [34, 35], it is possible to conclude from Figure 1(b) that, at least in aorta, kidney, and spleen, the EC of Tie2-*Scarb1* transgenic mice expressed significantly enhanced levels of the* Scarb1* transgene. Tissues, such as the liver and lung, which did not show a substantial increase in the expression of the transgene (Figure 1(b)) compared to the endogenous gene, are known to already have relatively high levels of endogenous* Scarb1* in nonendothelial cells [10].

### 3.2. Localization of SR-BI Protein in Endothelial Cells

**Figure 2:**
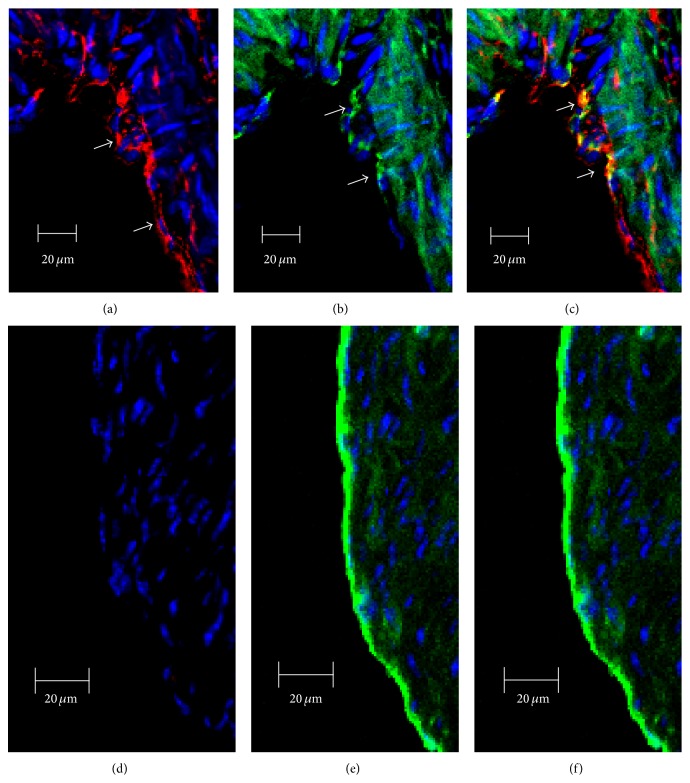
Immunofluorescent analysis of SR-BI localization in aorta of Tie2-*Scarb1* ×* Scarb1*-KO and* Scarb1*-KO mice. Mouse aorta sections were stained for SR-BI (a and d, red) and CD31 (b and e, green). Colocalization of SR-BI and CD31 was presented on (c) and (f). Blue = DAPI. (a, b, and c) Immunohistological analysis of SR-BI localization in aortic sections of Tie2-*Scarb1* ×* Scarb1*-KO mice. Colocalization of SR-BI and CD31 was found in aortic endothelial cells. In several EC stained for SR-B1 red color was present on both apical and basolateral sides (a). (d, e, and f) Immunohistological analysis of SR-BI localization in aortic sections of* Scarb1*-KO mice. There is no red signal in aorta from* Scarb1*-KO mice.

**Figure 3:**
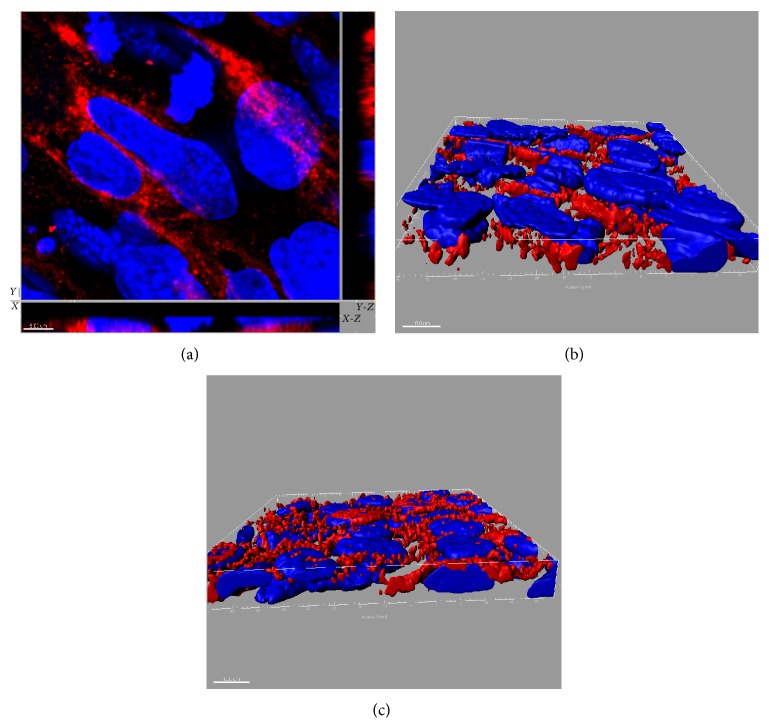
SR-BI distribution in polarized EC SVEC4-10 cells. SR-BI localization is presented in (a) as *x*-*y* image and orthogonal (*x*-*z* and *y*-*z*) views. (b) and (c) represent rendering of SR-BI staining (red) throughout the cells. Nuclei: blue. In (b) and (c) basolateral surface is on the top and the bottom, respectively. These results demonstrate the presence of SR-BI on both the apical and the basolateral sides of EC. The size of scale bar on (a) is 5 *μ*m and on (b) and (c) 10 *μ*m.

**Figure 4:**
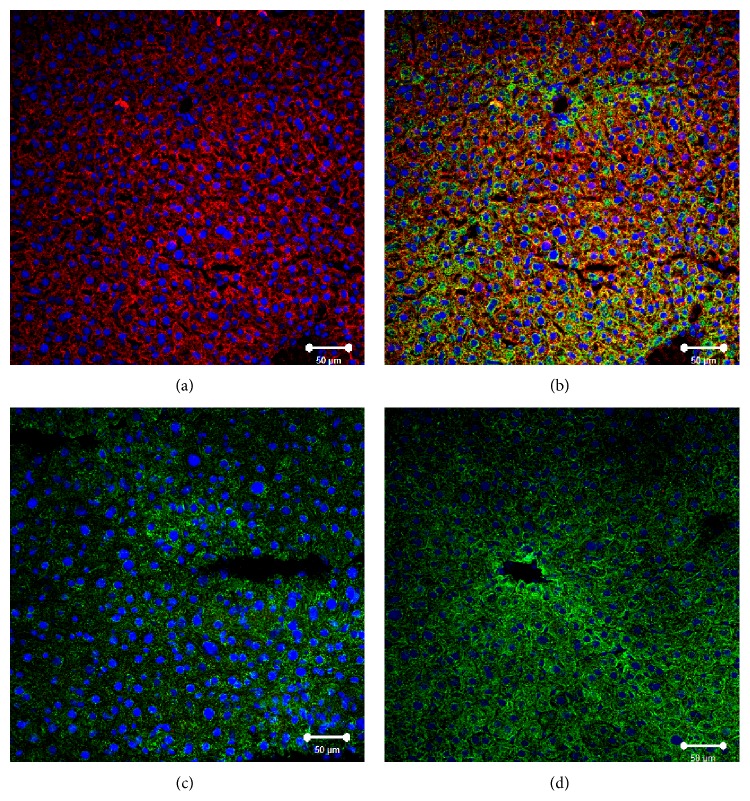
Immunofluorescent analysis of SR-BI localization in liver of normal, Tie2-*Scarb1* ×* Scarb1*-KO, and* Scarb1*-KO mice. Liver sections from normal (a, b) and Tie2-*Scarb1* ×* Scarb1*-KO mice (c) were stained for SR-BI (red) and cytokeratin 8–18 (green). Merge image for normal mouse (b) demonstrates strong presence of SR-BI in hepatocytes (b, yellow signal) and absence of detectable level of SR-BI protein in liver of Tie2-*Scarb1* ×* Scarb1*-KO mice (c). In* Scarb1*-KO mice there was no detectable level of SR-BI protein (d, staining for SR-BI). Blue = DAPI.

Immunofluorescent analysis revealed the presence of SR-BI in aortic EC of the Tie2-*Scarb1* ×* Scarb1*-KO mice but no expression was detected in* Scarb1*-KO mice (Figure 2). SR-BI was clearly located on both the apical and basolateral sides of endothelial cells in the aortas isolated from Tie2-*Scarb1* ×* Scarb1*-KO mice (Figures 2(a) and 2(c)). This finding was confirmed in experiments on polarized cultures of immortalized mouse SVEC4-10 EC using confocal laser-scanning immunofluorescent microscopy. SR-BI was detected on the apical surface but most of the SR-BI was detected between cells on the basolateral membrane and only limited staining was observed on the basal surface (Figure 3). To better understand the specificity of Tie2-*Scarb1* expression we used immunofluorescent analysis to examine the presence of SR-BI in liver of normal and Tie2-*Scarb1* ×* Scarb1*-KO mice (Figure 4). In hepatocytes of normal mice, we found abundant SR-B1, as expected (Figures 4(a) and 4(b)), whereas in liver of Tie2-*Scarb1* ×* Scarb1*-KO mice there was no detectable SR-BI protein (Figure 4(c)).

### 3.3. Effect of Endothelial Expression of SR-BI on Plasma Lipids

The increased expression of SR-BI from the Tie2-*Scarb1* transgene in EC had a limited effect on plasma lipids on both the C57Bl/6N and the apoE-KO backgrounds (Table 1). Compared to their sibling controls, the C57Bl/6N Tie2-*Scarb1* transgenic mice showed small 1.2- and 1.35-fold decrease in triglycerides and HDL cholesterol (HDL-C), respectively (*P* < 0.005). There were no statistically significant differences in the level of any plasma lipids between Tie2-*Scarb1* × apoE-KO and nontransgenic sibling apoE-KO control females. Similarly, no significant changes were observed in plasma lipids in Tie2-*Scarb1* ×* Scarb1*-KO mice compared to nontransgenic sibling* Scarb1*-KO mice.

**Figure 5:**
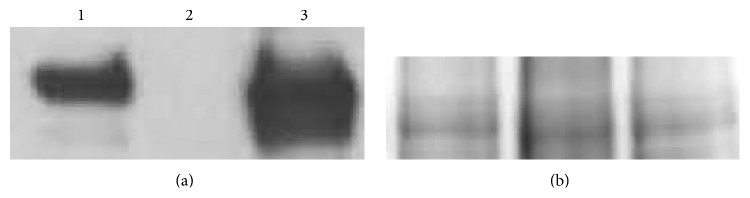
Western blot and Coomassie stain of membrane fractions isolated from livers of normal C57Bl/6N (lane 1),* Scarb1*-KO (lane 2), and LIV11-*SCARB1* ×* Scarb1*-KO (lane 3) mice. 20 *μ*g of membrane protein was loaded into each lane. (a) Western blot (anti-SR-BI antibody). (b) Coomassie stain (loading control for Western blot) encompassing the same MW region as SR-BI. Aliquots from the same tube were loaded for the Western blot and for the Coomassie-stained gel, which shows comparable loading between the three samples.

Given the importance of hepatic SR-BI in regulating plasma lipids and maintaining functional HDL [16, 36], we created two additional lines of mice in which SR-BI protein was expressed only in endothelial cells and/or in the liver. First, we generated a transgenic mouse line (LIV11-*SCARB1*), with the human* SCARB1* gene placed under control of the liver-specific LIV11 promoter [37]. By crossing this line, we then produced two other lines of mice: (1) LIV11-*SCARB1* ×* Scarb1*-KO (liver-only expression) and (2) Tie2-*Scarb1* × LIV11-*SCARB1* ×* Scarb1*-KO (EC and liver expression). Compared to C57Bl/6N mice, the expression of SR-BI protein in the liver was approximately 3-fold higher in LIV11-*SCARB1* ×* Scarb*1-KO mice when tested with an antibody that cross-reacts with both human and mouse SR-BI (Figure 5).

**Figure 6:**
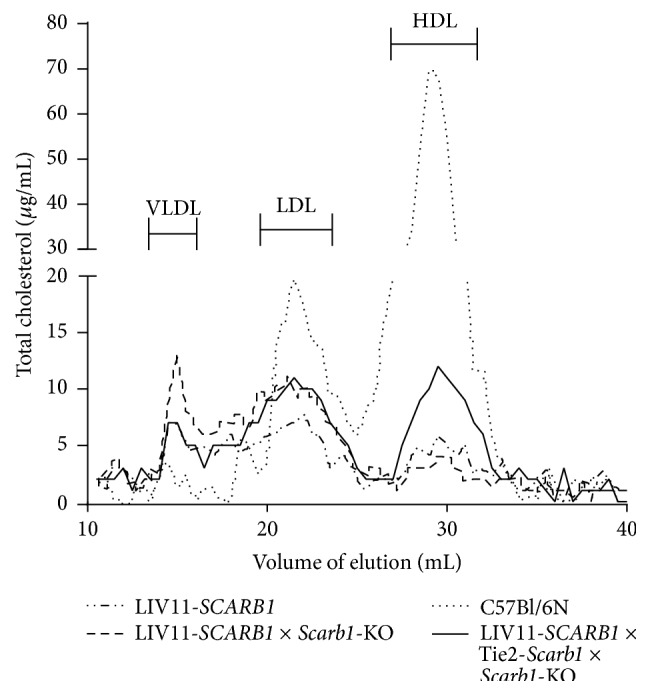
Effect of* Scarb1* expression in endothelium on distribution of total cholesterol between different lipoprotein fractions in plasma of transgenic females. The plasma from three different lines of transgenic females was compared: LIV11-*SCARB1* transgenic mice overexpressing* SCARB1* in liver with functioning endogenous* Scarb1*; LIV11-*SCARB1* ×* Scarb1*-KO (expressing SR-BI only in liver); and Tie2-*Scarb1* × LIV11-*SCARB1* ×* Scarb1*-KO (SR-BI was expressed in liver and endothelium). Normal C57Bl/6N female plasma was included as a control. In each case 350 *μ*L of plasma was collected from 4 to 6 females and fractionated by FPLC.

The effect of hepatic and EC expression of SR-BI on plasma lipids is shown in Table 2. The high level of hepatic expression of human SR-BI in LIV11-*SCARB1* transgenic mice significantly reduced by more than 5-fold total cholesterol and had a similar effect in reducing other plasma lipids, as has been previously described [16, 36]. By FPLC analysis (Figure 6), overexpression of SR-BI in the liver led to a marked depletion in plasma HDL-C, as well as a decrease in the cholesterol content of the other major lipoprotein classes. The LIV11-*SCARB1* ×* Scarb1*-KO mice had a similar level of plasma lipids and a similar FPLC profile as LIV11-*SCARB1* transgenic mice (Table 2 and Figure 6). Interestingly, the effect of combined endothelial and hepatic expression of SR-BI (Tie2-*Scarb1* × LIV11-*SCARB1* ×* Scarb1*-KO) was clearly visible when these mice were compared with LIV11-*SCARB1* ×* Scarb1*-KO animals: total cholesterol and other plasma lipids were significantly increased by 1.4–1.7-fold, and HDL-C levels were partially restored (Table 2 and Figure 6).

### 3.4. Effect of Endothelial SR-BI Expression on Atherosclerosis

**Figure 7:**
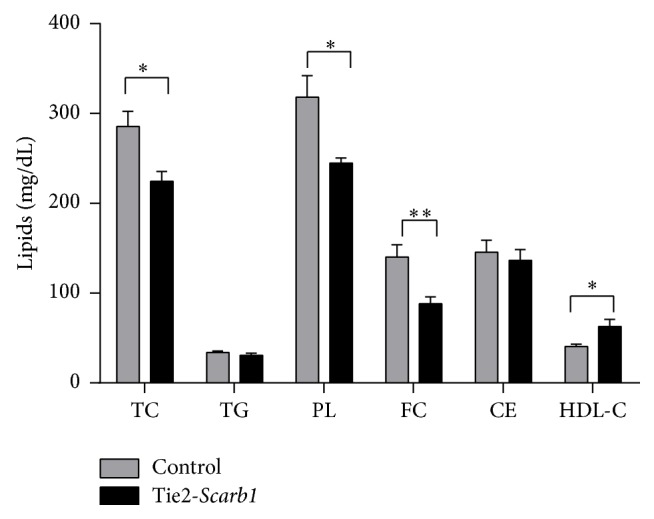
Effect of HFHC diet on plasma lipids in Tie2-*Scarb1* transgenic females in comparison with C57Bl/6N females. The analysis was done after 4.5 month of HFHC diet; 10 homozygous Tie2-*Scarb1* transgenic and 14 C57Bl/6N control females of similar age were used. Abbreviations for lipids are the same as in Table 1. ^*∗*^ *P* < 0.02; ^*∗∗*^ *P* < 0.008.

Tie2-*Scarb1* transgenic and C57Bl/6N control females were placed on a HFHC diet to assess the impact of endothelial SR-BI expression on atherosclerosis. In contrast to what was observed on a standard chow diet (Table 1), Tie2-*Scarb1* transgenic mice on the HFHC diet appeared to have a less proatherogenic lipoprotein profile than C57Bl/6N mice (Figure 7). Compared to C57Bl/6N control females, the Tie2-*Scarb1* mice had a significant (*P* < 0.01) reduction in total cholesterol (21 ± 6%), free cholesterol (37 ± 7%), and phospholipids (23 ± 6%). In addition, HDL-C was increased by 1.5 ± 0.2-fold (*P* < 0.02) in the Tie2-*Scarb1* mice. These atheroprotective lipoprotein differences between transgenic and control females were consistent with the* en face* analysis of aortic lesions. Representative images of aortic lesions, as determined by* en face* analysis, are shown in Supplementary Figure 1.

**Figure 8:**
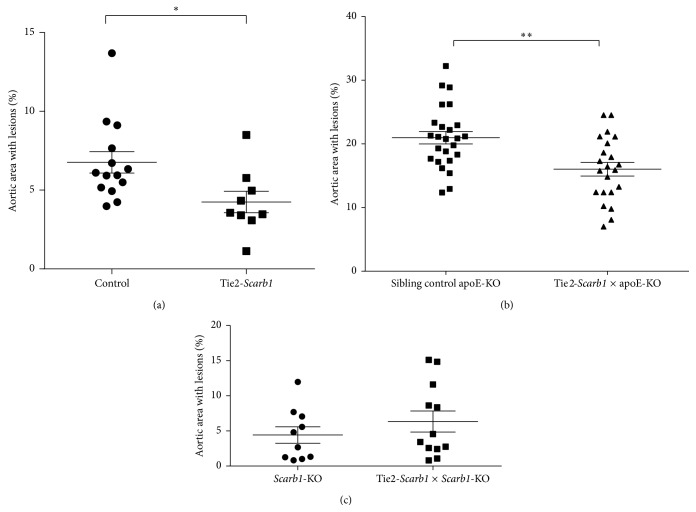
Effect of EC specific overexpression of* Scarb1* on atherosclerosis. Percent of aortic lesions, as determined by* en face* analysis, is shown (a) for Tie2-*Scarb1* transgenic females (*N* = 9) versus C57Bl/6N control females (*N* = 14) on HFHC diet for 6 months (^*∗*^ *P* < 0.02), (b) for 8-month-old Tie2-*Scarb1* × ApoE-KO mice (*N* = 22) versus nontransgenic sibling controls (*N* = 25) on normal chow diet (^*∗∗*^ *P* < 0.002), and (c) for Tie2-*Scarb1* ×* Scarb1*-KO mice (*N* = 12) versus nontransgenic sibling* Scarb1*-KO controls (*N* = 10) on HFHC diet for 3 months (*P* > 0.3).

When analyzed for atherosclerosis after 6 months on the HFHC diet, the Tie2-*Scarb1* transgenic mice had 37% less (*P* < 0.02) aortic surface lesions compared to C57Bl/6N control females (Figure 8(a)). Overexpression of SR-BI in endothelium was also able to decrease atherosclerosis when crossed with apoE-KO mice. The Tie2-*Scarb1* × apoE-KO females at 8 months of age had 24% less surface aortic lesions than control sibling females (*P* < 0.002) (Figure 8(b)). In contrast, when the Tie2-*Scarb1* transgenic mice were crossed with the* Scarb1*-KO animals and placed on the HFHC diet, the Tie2-*Scarb1* ×* Scarb1*-KO mice showed no significant difference in aortic atherosclerotic lesions compared to their* Scarb1*-KO sibling controls (Figure 8(c)). Similar to what was observed on the normal chow diet (Table 1), no major differences were also observed in these two lines in their plasma lipids on the HFHC diet (Figure 2 of the Supplemental Material).

### 3.5. HDL Transport and Cholesterol Flux in Endothelial Cell Culture Studies

**Figure 9:**
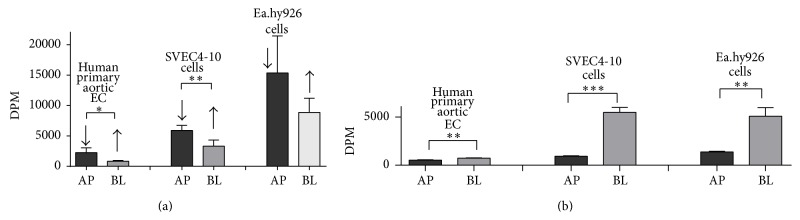
HDL transport and cholesterol flux in endothelial cell culture studies. (a) Penetration of HDL labeled by C^14^-cholesteryl oleate through polarized human primary aortic EC, SVEC4-10, and EA.hy926 EC after 4 hrs of incubation. AP-HDL was placed into Transwell (apical side of the cells); BL-HDL was placed into basolateral compartment. The concentration of labeled HDL in media was 50 *μ*g/mL (for protein). The specific activity of HDL was 2000 DPM/*μ*g. Arrows show direction of HDL penetration through the cells: from apical to basolateral side (arrow pointing down) or in the opposite direction (arrow pointing up). Dark bars represent results for loading HDL into top compartment, whereas light grey bars show results for placing labeled HDL in the bottom compartment. Results are presented as the mean ± 1 SEM. ^*∗*^ *P* < 0.02; ^*∗∗*^ *P* < 0.008. (b) Uptake of C^14^-cholesteryl ester from ^14^C-cholesteryl oleate-HDL by polarized human primary aortic EC, SVEC4-10, and EA.hy926 EC after 4 hours of incubation. Conditions of the experiment and bar labeling were the same as for (a). Results are presented as the mean ± 1 SEM. ^*∗∗*^ *P* < 0.006; ^*∗∗∗*^ *P* = 0.0001.

In order to better understand HDL transport and cholesterol flux by EC, experiments were performed on radiolabeled lipid transfer across polarized EC grown on tissue culture inserts, as well as cell uptake (Figure 9). Similar to what has been described for labeled free cholesterol on HDL [8, 9], ^14^C-cholesteryl oleate-labeled HDL placed either into apical or basolateral compartments was able to cross through the EC monolayer to the other side but there was more efficient transfer from the apical to the basolateral compartment (Figure 9(a)). FPLC analysis has shown that after transfer through EC, HDL particles were still close to their original size (data not shown). When ^14^C-cholesteryl oleate-labeled HDL was placed into either the apical or the basolateral compartment, cellular uptake and internalization of the labeled cholesteryl ester was also observed from both compartments, but the uptake was significantly higher from the basolateral than from the apical surface of EC. The difference was 1.4 ± 0.1-fold for human primary aortic EC and 3.7 ± 0.3- and 5.9 ± 0.7-fold for Ea.hy 926 and SVEC4-10 cells, respectively (Figure 9(b)). This observation led us to suggest that in aorta and other blood vessels, after penetration through a layer of EC into the interstitial space, HDL was able to acquire cholesterol from macrophages, to esterify it by LCAT associated with HDL and to deliver it back to the basolateral surface of the EC for selective uptake.

**Figure 10:**
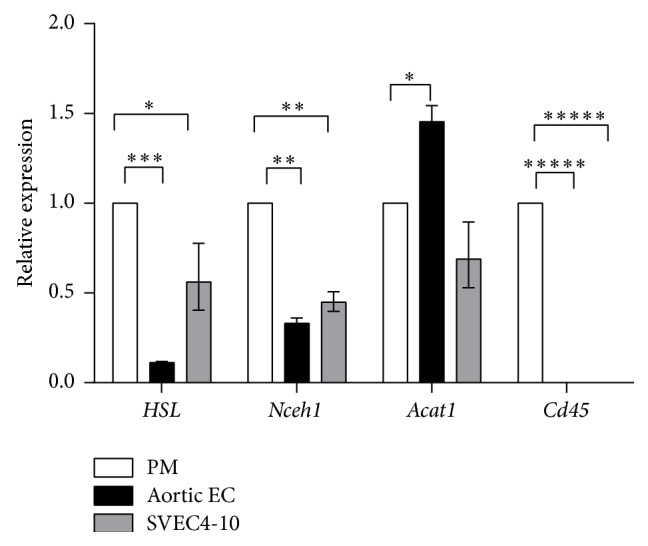
RT-PCR analysis of* Hsl*,* Nceh1*,* Acat1,* and* Cd45* gene expression in normal mouse aortic EC and SVEC4-10 relative to expression of the same genes in peritoneal macrophages (PM). Results are presented as the mean ± 1 SEM. ^*∗*^ *P* < 0.05; ^*∗∗*^ *P* < 0.001; ^*∗∗∗*^ *P* < 0.0001; ^*∗∗∗∗∗*^ *P* < 0.000001.

To determine if EC express genes that are known to be involved in intracellular processing of cholesterol [38], we checked mRNA levels of two neutral cholesteryl ester hydrolases,* Nceh1* and hormone sensitive lipase (*Hsl*), as well as the expression of acyl CoA cholesteryl acyltransferase 1 (*Acat1/Soat1*), in primary cultures of mouse aortic EC and in mouse SVEC4-10 EC, and as a control in peritoneal macrophages isolated from normal C57Bl/6N mice (Figure 10). Cultured EC were found to be free from any contamination with macrophages, as Cd45 expression was undetectable. Both mouse aortic EC and SVEC4-10 expressed* Nceh1* mRNA, but at about half the level observed in the macrophages. In addition, the two EC lines expressed* Hsl* mRNA at the level of 0.1–0.6 compared to macrophages. Aortic EC expressed* Acat1* mRNA at 1.5-fold higher level than peritoneal macrophages (*P* < 0.05); the difference between SVEC4-10 and macrophages in* Acat1* gene expression was not statistically significant.

## 4. Discussion

The main findings from this study are that increased SR-BI expression in EC can modulate lipid and lipoprotein levels and that it can protect against atherosclerosis in mice. It has been previously shown that extrahepatic expression of SR-BI can have favorable effects on plasma lipids and on the pathogenesis of atherosclerosis [17], but whether increased expression in EC is beneficial in these two processes was not previously known.

In both C57Bl/6N mice and apoE-KO mice on a normal chow diet, the increased expression of the* Scarb1* transgene in EC had only a small overall effect on plasma lipids (Table 1), which is probably due to the relatively high level of expression of the endogenous* Scarb1* gene in endothelium. We did consistently observe, however, a small decrease in plasma levels of triglycerides and HDL cholesterol in Tie2-*Scarb1* transgenic mice (Table 1). It is possible that SR-BI overexpression in EC helps to mobilize HDL for interaction with endothelial lipase, which could lead to increased catabolism of HDL and to decreased levels of triglycerides and HDL cholesterol in plasma. On the other hand, when Tie2-*Scarb1* transgenic mice were placed on a HFHC diet and their plasma total cholesterol was increased more than threefold (Table 1 and Figure 7), the additional expression of SR-BI in endothelium of Tie2-*Scarb1* transgenic mice stimulated the transcytosis of HDL from the luminal to the subluminal space and increased flux of the excess of cholesterol from peripheral tissue cells to the circulation by these HDL particles. The additional HDL-C in the transgenic mice on the HFHC diet was probably significantly higher than the effect of SR-BI transgene on decreasing HDL-C observed in mice on normal chow diet (Table 1 and Figure 7).

The inability of the Tie2-*Scarb1* transgene to modulate plasma lipids in* Scarb1*-KO mice is also not unexpected, given the known fact that hepatic expression of SR-BI is the main determinant of plasma lipoprotein levels [16, 36]. Even in macrophages, which are thought to be an important source of cholesterol removed by HDL in the RCT pathway, the increased expression of SR-BI or its complete absence in macrophages did not have a major effect on the level plasma lipids and HDL-C [18, 19]. We did observe, however, a significant effect of endothelial SR-BI expression on plasma lipids when mice were deficient for endogenous SR-BI but expressed human SR-BI in liver (compare Tie2-*Scarb1* × LIV11-*SCARB1* ×* Scarb1*-KO versus LIV11-*SCARB1* ×* Scarb1*-KO mice, Table 2). The expression of SR-BI in endothelium, in this case, was accompanied by a 36–76% increase in all measured plasma lipids (Table 2), including HDL-C (Figure 6). It is important to note that the Tie2-*Scarb1* transgene did not express detectable SR-BI in the liver (Figure 4), so the observed effects of this transgene on plasma lipids in Tie2-*Scarb1* × LIV11-*SCARB1* ×* Scarb1*-KO versus LIV11-*SCARB1* ×* Scarb1*-KO mice most likely resulted from gene expression of Tie2-*Scarb1* in endothelium of the peripheral tissues.

On the HFHC diet, the* Scarb1* transgene in EC lowered proatherogenic lipids and raised HDL-C (Figure 7), which may have contributed to the observed atheroprotective effect of the transgene (Figure 8(a)). It was previously shown that extrahepatic expression of* Scarb1* decreased inflammation by lowering the proinflammatory cytokine IL-6 in plasma when mice were placed on a high fat diet [17]. SR-BI has also been shown in EC to promote the uptake of alpha-tocopherol, which could blunt oxidation and inflammation from the increased uptake of oxidized lipoproteins from the high fat diet [39]. Another possible mechanism of atheroprotection by the Tie2-*Scarb1* transgene may relate to the known bioactive signaling molecule sphingosine 1-phosphate, which participates in many cardiovascular effects of HDL, including reduction of VCAM-1 and ICAM-1 expression by EC and protection against atherosclerosis (see review [40]). It has been suggested that SR-BI plays a significant role in HDL-S1P formation, supporting the optimal level of S1P in cells and anti-inflammatory effects of HDL-S1P in EC (see reviews [40, 41]).

Similar to the transgenic mice on C57Bl/6N background, the Tie2-*Scarb1* × apoE-KO mice had less atherosclerosis on a normal chow diet than apoE-KO mice, again indicating that endothelial expression of SR-BI is atheroprotective (Figure 8(b)). In contrast,* Scarb1*-KO mice with expression of SR-BI in EC (Tie2-*Scarb1* ×* Scarb1*-KO) placed on HFHC diet did not show less atherosclerosis than* Scarb1*-KO mice (Figure 8(c)). It may be that in order for SR-BI in EC to have a beneficial effect on atherosclerosis the presence of functional HDL in the plasma may be required. As has been previously described [14], in the absence of functional SR-BI, the ability of hepatocytes to take up cholesterol esters from plasma was seriously impeded, as was the reverse cholesterol transport pathway. The level of HDL-C increased approximately 3-fold in Tie2-*Scarb1* ×* Scarb1*-KO mice compared to the transgenic mice on C57Bl/6N background (Table 1), but the HDL that accumulates in these* Scarb1*-KO mice is known to be abnormal in both protein and lipid composition and also in function [14, 36].

**Figure 11:**
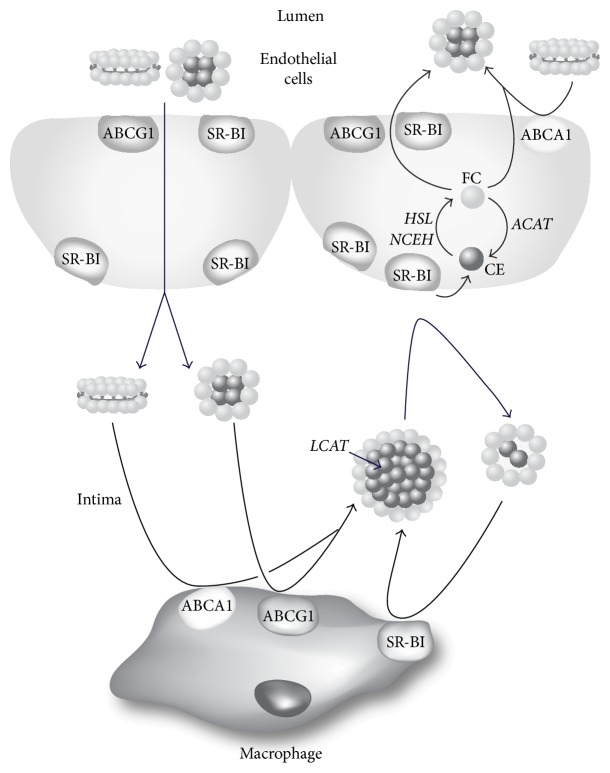
Model for intramural cholesterol flux by HDL. HDL from lumen penetrates EC, enters intimal space, and acquires cholesterol from macrophages through ABCA1, ABCG1, and SR-BI. After esterification by LCAT, cholesteryl esters are delivered to EC by HDL through interaction with SR-BI located in the basolateral membrane. After intracellular hydrolysis of cholesteryl esters by HSL or NCEH, free cholesterol is effluxed from EC by ABCA1, ABCG1, or SR-BI to HDL in the plasma compartment.

Our endothelial cell culture results (Figure 9) also provide another possible mechanism for the atheroprotective effect of SR-BI expression in EC. In experiments with polarized Ea.hy926 and SVEC4-10 cells we found that the ability of ^14^C-cholesteryl oleate-HDL to penetrate through EC from the apical surface was 2-3-fold higher than from the basolateral side. At the same time the cellular uptake of cholesteryl ester from HDL was more effective from the basolateral, rather than from the apical side (compare Figures 9(a) and 9(b)). It is generally believed that one of the main antiatherogenic effects of HDL is its ability to remove excess cholesterol from cells in the intima space of the vessel wall followed by its return by the lymphatics to the circulation, where it delivers its cholesterol to the liver for excretion [42, 43]. Based on the results of this study, an alternative possible pathway is shown in Figure 11. After acquiring cholesterol effluxed from arterial macrophages, HDL in the intimal space could possibly in a cyclical manner unload its cholesterol to endothelial cells through SR-BI. Smaller vessels, such as the coronary arteries in humans, do not have lymphatics [44], so this intravascular shuttling pathway could be important in maintaining cholesterol homeostasis. Once cholesteryl esters from HDL are taken up by SR-BI into EC, they could undergo hydrolysis by one of several possible cholesteryl ester hydrolysis enzymes, such as NCEH1 and HSL [38]. The newly generated intracellular free cholesterol could then be effluxed to the plasma compartment after the interaction of HDL in plasma with ABCA1 or ABCG1 transporters or SR-BI in the apical membrane of EC. This would be consistent with past findings showing that increased expression of both ABCA1 [10] and ABCG1 [45] in EC is atheroprotective. In favor of this suggestion is the dual localization of SR-BI in the basolateral and apical surfaces of EC (Figures 2(a), 2(c), 3(a), 3(b), and 3(c)). Consistent with this model, it was recently reported by Lim et al. (2013) that removal of cholesterol from peripheral tissues by lymphatic vessels was dependent on the uptake and transcytosis of HDL by SR-BI expressed on lymphatic endothelium [46]. Additional work, however, will be necessary to more fully examine each of the steps described in Figure 11.

In summary, the results from this study revealed that the expression of SR-BI by EC can alter plasma lipids, modify cholesterol trafficking, and decrease atherosclerosis in mice, demonstrating the importance of endothelium in the etiology of this disease.

## Supplementary Material

In Supplementary Material we provide information about primers and probes used for genotyping Tie2-Scarb1 and LIV11-SCARB1 transgenic mice, for analysis of the transgenes condition (hetero- or homozygous) and for expression analysis by TaqMan assays of several individual genes, mentioned in the paper. In order to show the effect of EC specific expression of Scarb1 on atherosclerosis representative images of aortic lesions in the Tie2-Scarb1 transgenic mice on normal, ApoE-KO and Scarb1-KO backgrounds, as determined by en face analysis were included in Supplementary Fig. 1. In Supplementary Figure 2 it was shown that without expression of Scarb1 gene in liver additional expression of Tie2-Scarb1 was not able to change the level of plasma lipids.

## Figures and Tables

**Table 1:** Plasma lipid profiles of Tie2-*Scarb1* transgenic females on normal C57Bl/6N, *Scarb1*-KO, or apoE-KO backgrounds. All animals were kept on normal chow diet.

Genotype	TC	TG	PL	FC	CE	HDL-C
	Normal C57Bl/6N background					
Tie2-*Scarb1*, *N* = 35–39	85 ± 2	66 ± 2^a^	174 ± 4	20 ± 1	65 ± 2	45 ± 3^b^
Sibling control, *N* = 13	86 ± 3	79 ± 3	178 ± 7	22 ± 1	63 ± 2	61 ± 3

	ApoE-KO background					
Tie2-*Scarb1*, *N* = 28–33	463 ± 17	131 ± 21	260 ± 7	127 ± 4	346 ± 12	N/A
Sibling control, *N* = 9–11	455 ± 24	160 ± 42	240 ± 9	124 ± 9	333 ± 21	N/A

	*Scarb1*-KO background					
Tie2-*Scarb1*, *N* = 15–19	232 ± 8	101 ± 6	316 ± 8	108 ± 7	124 ± 10	132 ± 6
Sibling control, *N* = 9–12	221 ± 7	105 ± 5	310 ± 14	104 ± 5	118 ± 9	103 ± 16

All values are expressed in units of mg/dL. ^a^ *P* < 0.005; ^b^ *P* < 0.0002. TC: total cholesterol; TG: triglycerides; PL: phospholipids; FC: free cholesterol; CE: cholesteryl ester.

**Table 2:** Plasma lipid profiles of normal C57Bl/6N, LIV11-*SCARB1* transgenic, LIV11-*SCARB1* × *Scarb1*-KO, and Tie2-*Scarb1* × LIV11-*SCARB1* × *Scarb1*-KO females. All animals were kept on normal chow diet.

Genotype	TC	TG	PL	FC	CE	HDL-C
C57Bl/6N females, *N* = 49–99	76 ± 1	80 ± 2	172 ± 4	15 ± 1	60 ± 1	50 ± 2
LIV11-*SCARB1*, *N* = 21–40	15 ± 1^a^	38 ± 3^a^	43 ± 4^a^	4 ± 1^a^	10 ± 2^a^	<3
Ratio control/LIV11-*SCARB1*	5.2 ± 0.5	2.1 ± 0.2	4.0 ± 0.4	4.0 ± 1.0	6.0 ± 1.0	

The effects of endothelial expression of *Scarb1* in *Scarb1*-deficient mice with LIV11-*SCARB1* in homozygous condition						
Tie2-*Scarb1* × LIV11-*SCARB1* × *Scarb1*-KO, *N* = 32–38	29 ± 2^b^	75 ± 10^c^	65 ± 3^b^	7 ± 1^d^	21 ± 1^e^	N/A
LIV11-*SCARB1* × *Scarb1*-KO, *N* = 28–32	20 ± 1	52 ± 4	45 ± 2	4 ± 0.5	16 ± 2	N/A
Ratio Tie2-*Scarb1* × LIV11-*SCARB1* × *Scarb1*-KO/LIV11-*SCARB1* × *Scarb1*-KO	1.5 ± 0.1	1.5 ± 0.2	1.4 ± 0.1	1.7 ± 0.3	1.4 ± 0.1	N/A

All values are expressed in units of mg/dL. Among the LIV11-*SCARB1* females, 17 were homozygous and 23 were heterozygous for the *SCARB1* transgene. LIV11-*SCARB1* × *Scarb1*-KO and Tie2-*Scarb1* × LIV11-*SCARB1* × *Scarb1*-KO females were homozygous for *SCARB1* transgene.

^a^ *P* < 0.0000001; ^b^ *P* = 0.0001; ^c^ *P* < 0.05; ^d^ *P* = 0.0005; ^e^ *P* < 0.004. Abbreviations for lipids are the same as in Table 1.
