# Supplementary material for: Endothelial Expression of Scavenger Receptor Class B, Type I Protects against Development of Atherosclerosis in Mice
Source: Biomed Res Int. 2015 Oct 4;2015:607120. doi: 10.1155/2015/607120 (PMC4609362; doi:10.1155/2015/607120)

Endothelial expression of scavenger receptor class B, type I (SR-BI) alters plasma lipids and protects against development of atherosclerosis in mice

Supplemental Material

Boris L. Vaisman[a], Tatyana G. Vishnyakova[b], Lita A. Freeman[a], Marcelo J. Amar[a], Stephen J. Demosky[a], Chengyu Liu[c], John A. Stonik[a], Maureen L. Sampson[d], Milton Pryor[a], Alexander V. Bocharov[b], Amy P. Patterson[b], and Alan T. Remaley[a]

[a] Lipoprotein Metabolism Section, Cardiovascular-Pulmonary Branch, National Heart, Lung, and Blood Institute, National Institutes of Health, Bethesda, MD
[b] Division of Diabetes, Endocrinology, and Metabolic Diseases, National Institute of Diabetes and Digestive and Kidney Diseases , National Institutes of Health, Bethesda, MD
[c] Transgenic Core, National Heart, Lung, and Blood Institute, National Institutes of Health, Bethesda, MD
[d] Dept. of Laboratory Medicine, Clinical Center, National Institutes of Health, Bethesda, MD

Primers and probes used for genotyping.

For genotyping Tie2-*Scarb1* and LIV11-*SCARB1* transgenic mice and distinguishing heterozygous from homozygous mice, quantitative real-time PCR was used. TaqMan assays were supplied by Life Technologies or designed using their Primer Express 3.0 software.

Two separate assays were used to verify the presence of the Tie2-*Scarb1* transgene. First, a TaqMan assay that specifically recognized the promoter region of the Tie2-*Scarb1* transgene but not the endogenous mouse Tie2 promoter was designed by including, in the reverse primer, the sequence for the Not I site present in the transgene but absent from the endogenous mouse Tie2 gene. In addition, the presence of the Tie2-*Scarb1* transgene was confirmed by normal PCR with forward primer GGTGTTTCTCCTTGCCGCCA, located in the Tie2 promoter region, and reverse primer AACGCCGAGCGCAGCAAACA, located in mouse *Scarb1* cDNA.

Genotyping of LIV11-*SCARB1* mice was carried out using an ABI TaqMan assay for the human *SCARB1* gene (see Supplementary Table 1).

Scarb1-KO and *apoE*-KO alleles were confirmed by PCR as recommended by Jackson Laboratories for *Scarb1*-KO (stock # 003379) and *ApoE*-KO (stock 002052) mice.

Supplementary Table 1. Life Technologies TaqMan assays used for expression analysis of the transgenes or mouse genes or for mouse genotyping

Gene/Symbol/Aliases	Assay ID number
Human scavenger receptor class B, member 1/*SCARB1*	Hs00969821_m1
Eukaryotic *18S rRNA*	4319413E
Protein tyrosine phosphatase, receptor type, *C/Ptprc/Cd45*	Mm01293575_m1
Lipase, hormone sensitive/*Lipe*	Mm00495359_m1
Arylacetamide deacetylase-like 1 or Neutral cholesteryl ester hydrolase /*Nceh1*	Mm00626772_m1
Scavenger receptor class B, member 1/*Scarb1*	Mm00450226_m1
Mouse *Soat1/Acat1*	Mm00486279_m1
Mouse actin, beta/*Actb*	4352341E
Mouse *28S rRNA*	Forward primer: TCTGCCCAGTGCTCTGAATG; fluorogenic TaqMan probe with FAM and quencher: CAAAGTGAAGAAATTC; reverse primer: CGTTTACCCGCGCTTCAT
Tie2-*Scarb1* (for genotyping only)	Forward primer: CCATGCGAGCGGGAAGT; fluorogenic TaqMan probe with FAM and quencher: AGTCAACAACTCACAACTTTG; reverse primer: CGGCCGCCAAAGCTTAC

When expression of *18S* or *28S rRNA* reference genes was measured, the concentration of cDNA in the qPCR wells was decreased by 512-fold in comparison with test samples to match the level of expression.

Supplementary Figure 1. Effect of HFHC diet on plasma lipids in Tie2-*Scarb1*-KO x *Scarb1*-KO mice compared to *Scarb1*-KO littermate control.

13 female Tie2-*Scarb1* x *Scarb1*-KO mice and 9 female *Scarb1*-KO sibling controls were placed on a HFHC diet for 3 months and plasma lipids were then analyzed. The following abbreviations were used for mice: KO – *Scarb1-KO*, Tr-gene+KO – Tie2-*Scarb1* x *Scarb1*-KO. Abbreviations used for lipids: TC – total cholesterol; TG – triglycerides; PL – phospholipids; FC – free cholesterol; CE – cholesteryl ester.

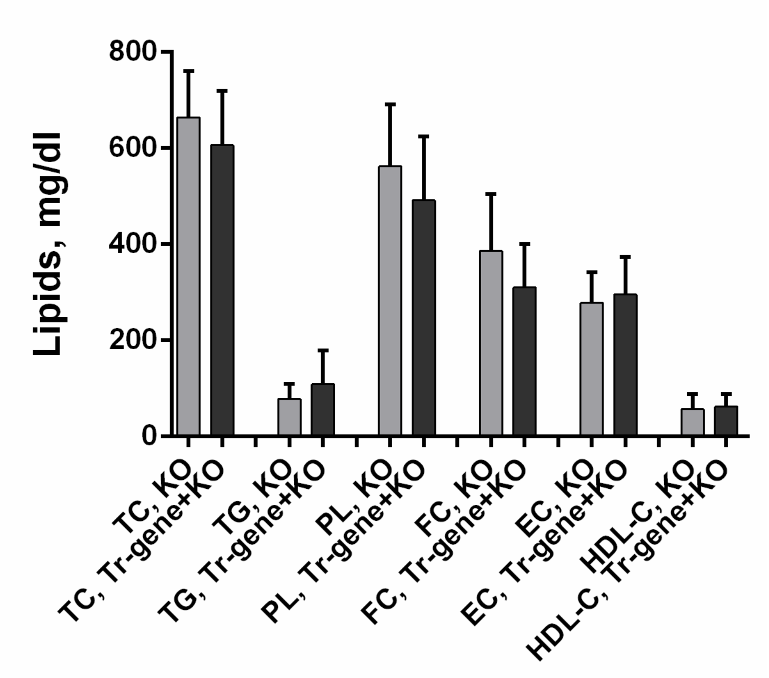

There were no statistically significant differences between these two groups.

Supplementary Figure 2. Effect of EC specific expression of *Scarb1* on atherosclerosis.

Representative images of aortic lesions, as determined by *en face* analysis, is shown.

Panel A. Anti-atherogenic effect of Tie2-*Scarb1* gene on development of atherosclerosis in Tie2-*Scarb1* transgenic and normal C57Bl/6N females after 6 months on Paigen high fat high cholesterol diet.

Panel B. Protective effect of Tie2-Scarb1 transgene against development of atherosclerosis in apoE-deficient mice. Shown aortic lesions developed in 8 months old

Tie2-*Scarb1* x apoE-Ko female and non-transgenic sibling apoE-Ko control kept on normal chow diet.

Panel C. Aortic lesions in Tie2-*Scarb1* x *Scarb1*-KO females and in non-transgenic sibling *Scarb1*-KO females developed after 3 months on HFHC diet. On *Scarb1*-knockout background Tie2-*Scarb1* transgene was not able to protection against development of atherosclerosis.

A

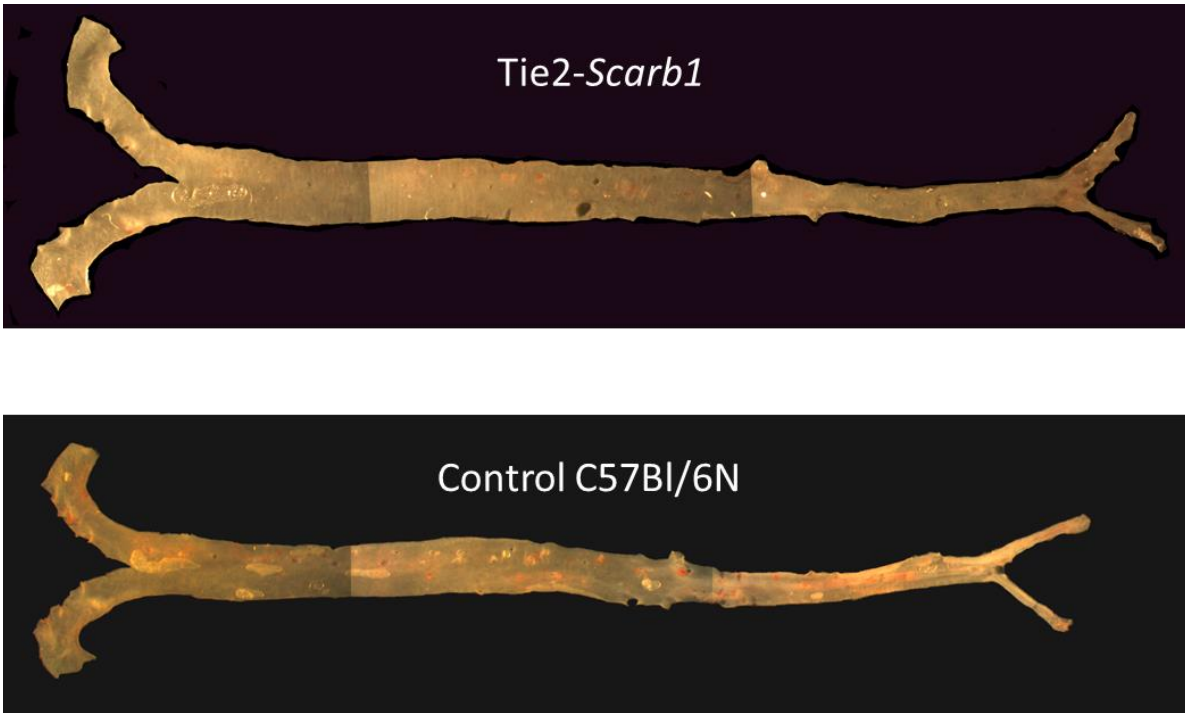

B

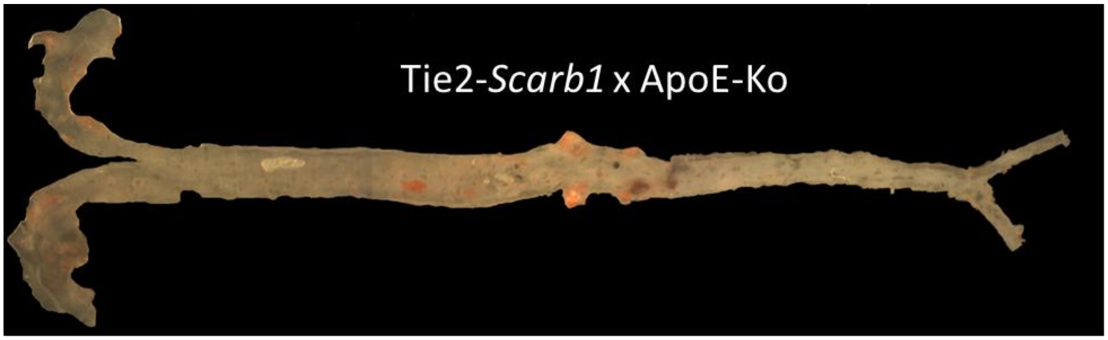

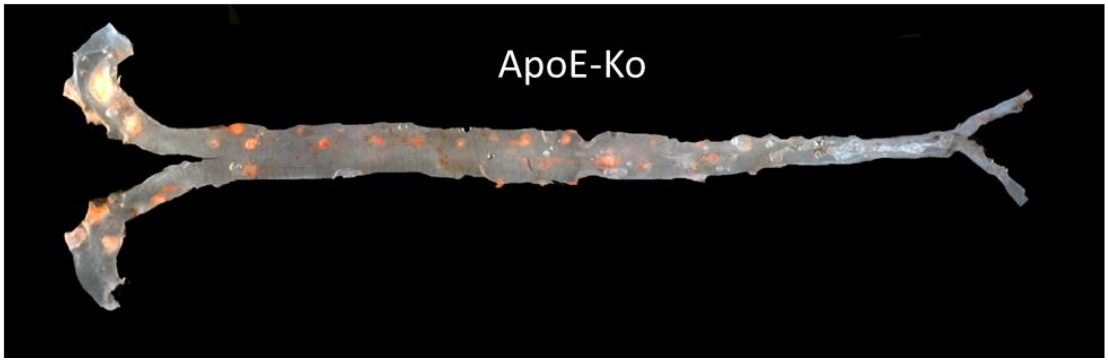

C

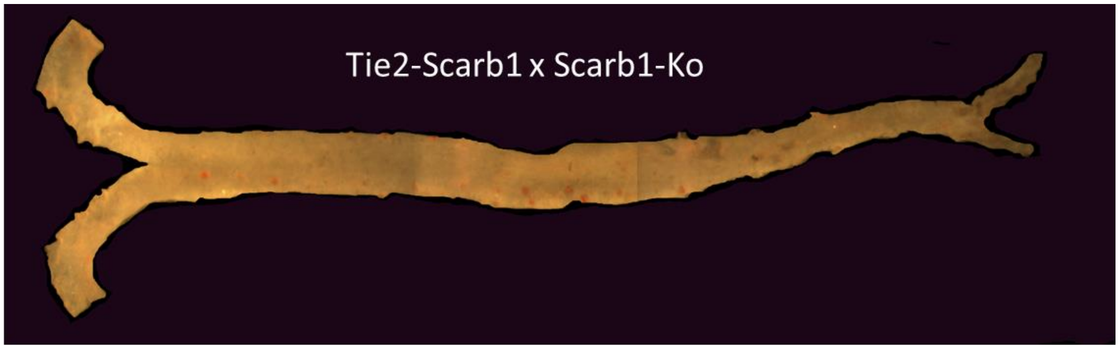

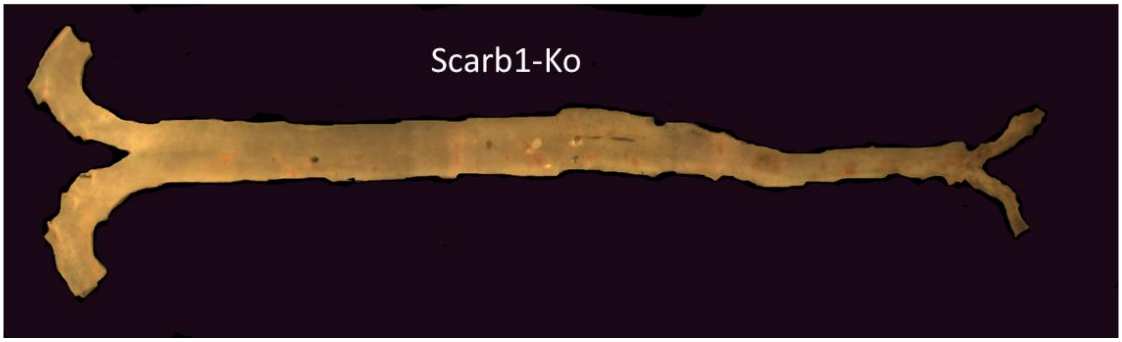

Supplement: Supplementary file 1 — In Supplementary Material we provide information about primers and probes used for genotyping Tie2-Scarb1 and LIV11-SCARB1 transgenic mice, for analysis of the transgenes condition (hetero- or homozygous) and for expression analysis by TaqMan assays of several individual genes, mentioned in the paper. In order to show the effect of EC specific expression of Scarb1 on atherosclerosis representative images of aortic lesions in the Tie2-Scarb1 transgenic mice on normal, ApoE-KO and Scarb1-KO backgrounds, as determined by en face analysis were included in Supplementary Fig. 1. In Supplementary Figure 2 it was shown that without expression of Scarb1 gene in liver additional expression of Tie2-Scarb1 was not able to change the level of plasma lipids. [file 607120.f1.pdf]
